# Build Your Own Microscope: Step-By-Step Guide for Building a Prism-Based TIRF Microscope

**DOI:** 10.3390/mps1040040

**Published:** 2018-11-03

**Authors:** Dalton R. Gibbs, Anisa Kaur, Anoja Megalathan, Kumar Sapkota, Soma Dhakal

**Affiliations:** Department of Chemistry, Virginia Commonwealth University, 1001 West Main Street, Richmond, VA 23284, USA; gibbsdr@vcu.edu (D.R.G.); kaura2@vcu.edu (A.K.); megalathana@vcu.edu (A.M.); sapkotak@vcu.edu (K.S.)

**Keywords:** fluorescence microscope, pTIRF, single-molecule FRET, single-molecule detection

## Abstract

Prism-based total internal reflection fluorescence (pTIRF) microscopy is one of the most widely used techniques for the single molecule analysis of a vast range of samples including biomolecules, nanostructures, and cells, to name a few. It allows for excitation of surface bound molecules/particles/quantum dots via evanescent field of a confined region of space, which is beneficial not only for single molecule detection but also for analysis of single molecule dynamics and for acquiring kinetics data. However, there is neither a commercial microscope available for purchase nor a detailed guide dedicated for building this microscope. Thus far, pTIRF microscopes are custom-built with the use of a commercially available inverted microscope, which requires high level of expertise in selecting and handling sophisticated instrument-parts. To directly address this technology gap, here we describe a step-by-step guide on how to build and characterize a pTIRF microscope for in vitro single-molecule imaging, nanostructure analysis and other life sciences research.

## 1. Introduction

**Figure 1:**
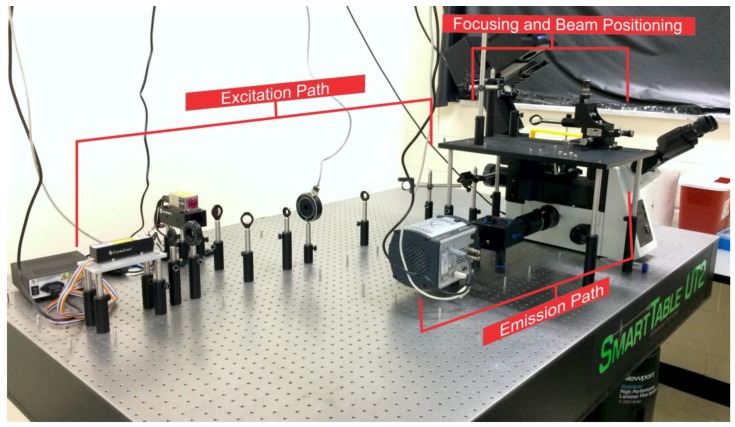
Prism-based total internal reflection fluorescence microscope (pTIRF) as it appears on the Smart Table UT2 (Newport Corporation, Irvine, CA, USA). The excitation path, focusing and beam positioning area, and the emission path are highlighted.

**Figure 2:**
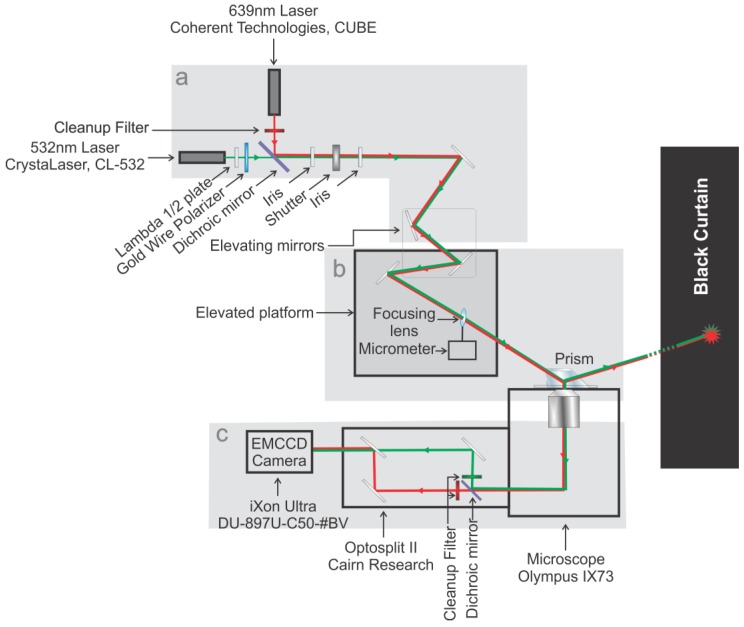
Schematic diagram of the pTIRF microscope. The entire setup is split into three parts: (**a**) the excitation path; (**b**) the focusing and beam positioning section, containing an elevated section just above the microscope stage; and (**c**) the emission path. Green and Red lines represent 532 nm and 639 nm lasers.

In this protocol we will start with the microscope layout and provide details on how to assemble the microscope parts. Then we will discuss laser alignments, enabling computer control, fluorescence imaging, data processing and safety. With this protocol, we hope to make the instrument assembly simple for future users of prism-based total internal reflection fluorescence (pTIRF) microscopy. Although objective-type TIRF microscopes are now commercially available, custom-built pTIRF instruments not only offer a higher signal to noise (S/N) ratio but also allow an easier manipulation and setup of the incident excitation beam(s), providing a greater experimental flexibility and allowing various biophysical studies [1,2]. While the working principle of the pTIRF and single molecule fluorescence resonance energy transfer (smFRET) has been discussed in detail in many publications [3,4,5,6,7,8,9,10], resources and protocols for the assembly of the instrument are very scattered and incomplete. The primary components of this microscope (lasers, optics, inverted microscope, and electron multiplying charge coupled device (EMCCD) camera, Figure 1) are common in scientific instrumentation. The layout of the instrument is detailed in Figure 2 and should be “read” from laser to camera. The instrument is composed of three general sectors: excitation path (Figure 2a); focusing and beam positioning (Figure 2b); and emission path (Figure 2c). We will walk through each of these sections in sufficient detail to explain the logic of component selection, ordering of these parts, assembly, and characterization. All of the microscope parts along with the part description and the quantity needed are compiled in Table 1 for a straightforward assembly of the microscope.

### 1.1. Excitation Light Source and Filters

**Figure 3:**
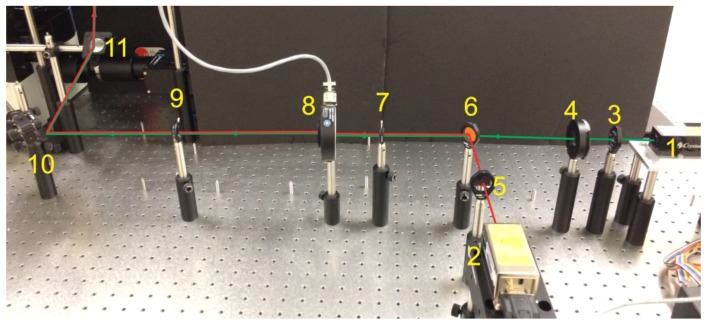
Real image of the excitation path of a pTIRF microscope corresponding to the “part a” of Figure 2. Red and green lines show the path of the lasers. (1—green laser, 2—Red laser, 3—half-wave plate, 4—polarizer, 5—clean-up filter, 6—dichroic mirror, 7—iris, 8—shutter, 9—iris, 10—mirror, 11-elevating mirror).

**Figure 4:**
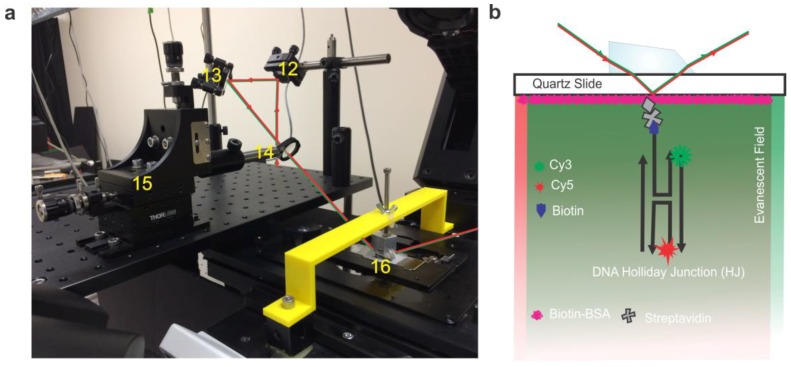
(**a**) Real image of the focusing and beam positioning section of a pTIRF microscope. Red and green lines show the path of the lasers. (12—mirror, 13—mirror, 14—focusing lens, 15—micrometer, 16—prism. The prism is mounted on the clamp that is attached to the prism support-arm (yellow). (**b**) Schematic of the smFRET setup with DNA Holliday junction (HJ) bound to the surface of a flow cell. An evanescent wave is created at the quartz/buffer interface by total internal reflection of lasers when passing through the prism (see Technical Notes for detail).

The instrument starts at its light sources (typically two of them per user’s choice, Figure 2a and Figure 3). These lasers serve to excite the fluorophores for later imaging. The first light source is a green (532 nm) laser with a lambda ½ wave plate, which allows for adjustment of polarization when needed [11], and a polarizing filter that serves as a power regulator as this source has no inbuilt power regulation. The second source is a red (639 nm) laser with a cleanup filter to remove any errant light created as a byproduct of laser generation in a range of 630–650 nm (Figure 3). These excitation lasers are mounted 90° to one another with the laser paths directed toward a dichroic mirror, which combines the two laser paths by transmitting green and reflecting red wavelengths. This dichroic mirror also serves as a cleanup filter for the green laser as it cuts out light above 565 nm. From this point, the now overlapping laser paths are directed through two irises separated ~30 cm to assist in focusing the instrument. A remotely controlled shutter is placed in the laser path allowing the excitation beam to be shuttered by the operator. The beam then strikes a series of mirrors designed to raise the beam onto an elevated platform. This section serves to generate, cleanup, and co-localize our excitation beam for focusing and beam positioning (Figure 4).

### 1.2. Focusing and Beam Positioning

In the focusing and beam positioning portion of the instrument (Figure 4a) we follow the beam onto an elevated platform positioned ~3 inches higher than and just to the left of the microscope stage. Mirror #11 in (Figure 3), which is located vertically below mirror #12 (Figure 4a), is used to reflect the laser beam at 90° to the elevated platform where the final mirror in this section directs the laser through the focusing lens into the prism (Figure 4a). The focusing lens is mounted on a three-axis micrometer, which allows ~1 cm of movement in the x, y, or z directions, thus allowing for fine adjustments to direct the focus of the laser overtop of the objective. A focusing lens with a 200 mm focal length (roughly the distance from the mounting point of the lens to the microscope objective) is used to focus the laser more intensely in the usable experimental area. The prism is held in position by a clamp attached to a support arm mounted to the microscope body (see Microscope Assembly section below for detail). This allows total internal reflection of the incident beam, thus producing an evanescent field on the quartz-buffer interface (Figure 4b). It is important to note that a non-fluorescent oil with a refractive index matching that of the quartz slide is used between the prism and the quartz slide.

### 1.3. Emission Path

**Figure 5:**
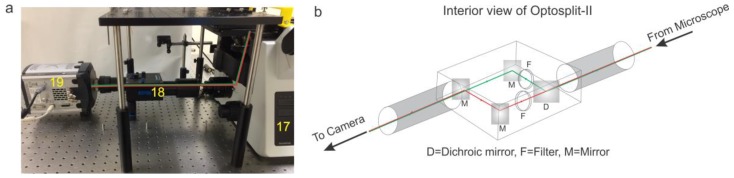
(**a**) Real image of the emission path of a pTIRF microscope. Red and green lines show the path of the lasers (17—Microscope, 18—Optosplit-II, 19—EMCCD Camera). (**b**) Diagram of Optosplit-II detailing the path of the light coming from the microscope and directed through a dichroic mirror (D), set of filters (F) and a series of mirrors (M) that serve to separate green and red emissions and parallelize the light to allow two color channels to be recorded on one EMCCD camera.

The fluorescence emission is captured by an inverted microscope objective (Olympus UPLSAPO 60 × W) and directed to the Optosplit-II (commercially available from Cairn (Faversham, UK)) (Figure 5). In the Optosplit-II, the incoming fluorescence emission is separated using a dichroic mirror and a series of mirrors into two beams, one red and one green. These beams are directed through cleanup filters to isolate the light from fluorescent signals and then parallelized and directed into the EMCCD camera [4] where they are fed into a computer for processing (Figure 2c).

## 2. Space Design

Space considerations are necessary before setting up a pTIRF system. It is recommended that a 6 × 4 feet space with easy access to electric outlets should be blocked off for positioning of the vibration isolation table, such as the 1200 mm × 1800 mm × 203 mm tune damper UT2 smart table from the Newport company (Irvine, CA, USA), leaving at least a 2 feet gap along the perimeter for easy clearance access all around the table. A metal rack with installed electrical outlets built and suspended from the ceiling roughly 6 ft above the floor is ideal to provide space for power supplies. Alternatively, a wall-mounted shelf or small table under the isolation table would also suffice. On the table, the microscope alone will take up about 19 × 22 inches of space on the breadboard, leaving the rest for positioning of the optics and camera. Extra space in close proximity to the laser table should be designated for a computer table with room for the PC, monitor, keyboard, and mouse. Ideally the computer table should be situated near the eyepiece of the microscope for ease of operation. Some amount of bench space may also be necessary for sample preparation and storage of materials. Two 5 × 2 feet tables, one designated for the computer set up and the other for extra bench space can provide sufficient room for extra work space. The dimensions we suggest leave ample room for additional optics to be added as necessary for future experiments. However, it should be noted that it is certainly possible to condense the pTIRF microscope set up to a 4 × 3 feet vibration isolation table. Even greater area can be conserved if creative optics solutions are utilized (e.g., fiberoptic runs).

Safety considerations must be taken while planning a space for the microscope. The microscope area must be completely enclosed to avoid any laser hazard and the stray room-light from reaching the camera during fluorescence measurements. Any open space such as windows or doorways should be blocked off using a black laser curtain to avoid accidental injury from scattered or reflected laser light. While in use, a “Laser in Use” sign is recommended to make anyone outside of the laser area aware to take necessary precautions before entering the area. While in the area, laser safety goggles should always be worn by all personnel (see Safety Considerations Section below).

## 3. Safety Considerations

It is very important to enclose the laser area to avoid safety hazards and keep everyone safe, thus it is necessary to:

- Block the surroundings using black laser curtains.
- Use the sign ‘laser in use’ to warn outsiders so that they may take necessary precautions before entering the area.
- Wear the appropriate laser safety goggles when using the laser and entering the laser area.
- Keep the shutter closed when the laser source is not required.
- Never look directly into the light path when the shutter is open.

## 4. Microscope Assembly

### 4.1. Optics Installation

**Figure 6:**
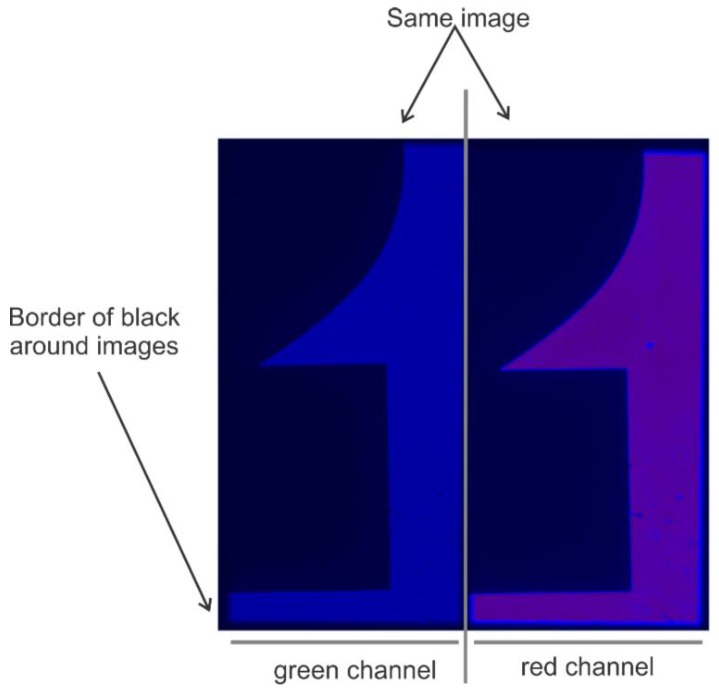
Image of a properly adjusted optosplit. Image of silkscreen printed “2” on glass slide was captured using the program called Single.exe (see Data Acquisition section for details). Note that if one uses Single.exe to acquire smFRET data, the green channel must be on the left and the red channel on the right. For proper alignments of the channels, the image size is adjusted to take up approximately half of the available space and that the images are well separated with a black border running around and between them.

1. Assemble optics on the laser table in a straight line along a single plane as specified in the instrument optical diagram.
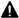
**CRITICAL STEP** Later steps will require that the lasers are aligned such that the beams are traveling co-linear. Ensuring that the lasers, emitters, and optics are mounted level with one another can prevent headaches down the line.
2. Build the elevated platform 90° to the end of the optical path next to the area intended for the microscope.
3. The lasers must be adjusted so that both beams pass through both irises. The iris closest to the laser is first narrowed, taking care not to close the iris completely, and the lasers readjusted so that both beams pass through its center. This step is then repeated with the second iris which is narrowed and the lasers adjusted so that the beams now pass through the center of both irises [12].
  **Note:** This process may take several repeated steps of narrowing one or both irises and adjusting the lasers to achieve total centering of the beams. Fine tuning of the leveling can be accomplished by allowing the lasers to leave the laser table and making minute adjustments to ensure that the points cast by both lasers hit at the same point on a wall. From this point onwards, the optics should not be adjusted unless something comes out of alignment.
4. Position the first mirror to direct the beam toward the elevated platform. The next two mirrors are placed in the line of the laser at roughly 45 degrees relative to the plane of the table and used to guide the beam toward the elevated stage and lift it to a level above the micrometer. The final mirror is used to direct the laser path through the focusing lens with 200 mm focal length (mounted on the micrometer) and into the prism, all adjusted so that the laser path enters into the prism at the appropriate angle to induce total internal reflection (in the case of our setup ~35°, calculated according to method detailed below in the Technical Note section).
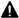
**CRITICAL STEP** It will be necessary to adjust the mirrors such that the focus is not elongated.
5. The microscope should be situated to the right of the elevated platform in such a way that the laser can be aimed through the optics and still maintain both the angle of total internal reflection and be within the focusing distance of the focusing lens.
6. The prism should be mounted in place over the flow cell by a clamping device affixed to the support arm which is screwed onto mounting hardware fixed to the upper body of the microscope.
  **Note:** Our prism clamp and support arm were custom manufactured by machining of aluminum and 3D printing respectively (see Technical Note section below for details). For a given microscope, the dimensions of the support arm and relative height of the prism will change, however the central principle of the design remains the same: to fix the position of the prism directly over the objective. So long as this is accomplished the design can be modified in many ways to accommodate various microscope brands and configurations.
7. Install the Optosplit-II (Carin Research, UK) into the imaging port and install the dichroic mirror and filters cube into the Optosplit-II. The aperture of the optosplit can then be tuned using the aperture adjustors to size the incoming light down to fit onto half of the EMCCD camera’s field of view and adjusted to split the channels to show parallel images (see Figure 5 and Figure 6). This can be accomplished by using a slide with some mm sized details (such as etched writing) to align the two channels in a parallel fashion for analysis by some smFRET program.
  **Note:** The Optosplit-II manual contains a more in-depth description of this process.
8. Install Single.exe, a program that is made available by the TJ Ha group designed to record single molecule fluorescence data (see Data Acquisition and Analysis section below. Instructions on how to configure the EMCCD camera can be found in the Single.exe reference manual but can be summarized as finding the appropriate “atmcd32d.dll” file for the selected camera model and overwriting the existing file in the Single.exe program directory. This dll can be found in the driver install software package that accompanies Andor cameras (https://cplc.illinois.edu/software/).
  **Note:** In all cases we used a ×64 Windows PC to run this software. OSX or Linux versions may not be compatible/available for all mentioned software.
9. **OPTIONAL STEP** Set up computer control of one or both lasers. In our case, we used a 639 nm Coherent CUBE Diode Laser (part#1069417) which comes equipped for computer control with an accompanying computer program Coherent Connection (http://cohrdownloads.blob.core.windows.net/file/CUBE%20Connection.zip). This laser’s serial cable connection was routed to our modern computer using a serial to USB connector.
  **Note:** When connecting an item to the processing computer with a serial cable, some configuration may be necessary to ensure that the coherent connection is monitoring the same serial port that the USB adaptor is feeding to.

### 4.2. Laser Alignment and Focusing

The alignment process, as outlined in the microscope assembly section, should roughly focus the laser onto the flow cell through the prism, but the largest portion of the laser alignment is the fine tuning.

1. The first step in fine tuning the laser alignment is to ensure that the laser lands immediately over the microscope objective. This can be aided by using a small piece of Scotch magic tape (preferably white in color) affixed on a glass slide to more easily visualize the location of the focus.
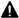
**CRITICAL STEP** It is essential to get the beam as close to the center of the objective lenses as possible as this will save time in adjusting the micrometer later on.
2. A flow cell filled with water should be placed onto the microscope, a drop of immersion oil should be placed on the top surface of the flow cell and the prism support arm assembly screwed into place.
  **Note:** It is important that the flow cell be assembled using a quartz slide as using glass will result in an unusable background signal.
3. The micrometers should be used to adjust the position of the focus on the X and Y axis until it is centered in the field of view through the eyepiece in the lowest objective. Once done, the Single.exe program can be used to track the intensity of the light coming off the focus.
4. The Z-axis is then adjusted either up or down to increase the intensity of the light, during which some X and Y adjustment needs to take place to keep the focus at the center of the field of view. This Z axis adjustment should be carried out until the intensity of the signal reaching the camera is at its maximum.
  **Note:** The center of the microscope field of view may be offset from the cameras field of view, so adjustments should be made accordingly.

## 5. Flow Cell Design and Construction for pTIRF Experiments

### 5.1. Cleaning Procedure

The cleaning procedure was followed from a published protocol [13].

1. Briefly, wash the slides in warm soapy water and then scrub thoroughly with a thick paste of Alconox, follow by rinsing the slides in deionized water, acetone, and ethanol successively.
2. Then flame the slides for 30 s on each side using a propane torch and immediately transfer them to a boiling base-piranha bath (Solution of 4% hydrogen peroxide and ammonia) for about 15 min and flame again on each side for 30 s with propane torch.

### 5.2. Design the Flow Cell

**Figure 7:**
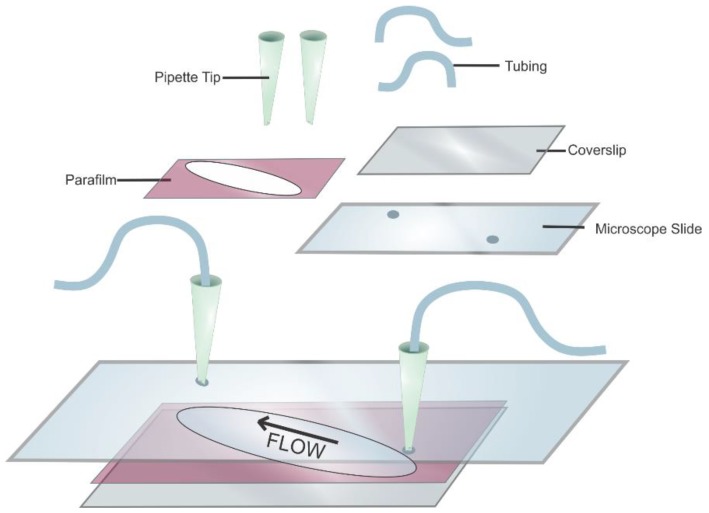
Schematic of a flow cell. The quartz microscope slide is shown with pipet tips and tubing to allow for buffer exchange. The sample chamber consists of a parafilm sandwiched between a microscope slide and the glass coverslip. The arrow shows the direction of the buffer flow.5.3. Surface-Functionalization of Flow Cell.

1. Take a pre-cleaned standard quartz slide (75 × 26 × 1 mm) with two diagonally drilled holes (drilled using a diamond-coated drill bit (1 mm in diameter) in a Dremel multitool purchased from Walmart, Bentonville, AR, USA) and add parafilm overtop (Figure 7).
2. Create a sample chamber by cutting the parafilm diagonally to encompass the drilled holes.
3. Cover the sample chamber with a glass coverslip (24 × 60 mm, Fisher Scientific, Waltham, MA, USA) and heat the whole assembly to 120 °C for 5 min on a hot plate in order to melt the parafilm, thus sealing the glass coverslip to the microscope slide.
4. Cut two 200 µL plastic pipette tips to about an inch long, insert into the holes and plume with tubing (0.02 in. ID, 0.06 in. OD, Cole-Palmer, Vernon Hills, IL, USA) using Double Bubble Quick-Set epoxy from Hardman Adhesives.
  **Note:** The physical obstructions of the pluming in this flow cell design limit the usable space for the experiment to ~1/5 of the slides total surface area.

1. Functionalize the flow cell by sequential incubation of 1 mg/mL biotinylated BSA and 0.2 mg/mL streptavidin for 5 min and 2 min, respectively [14].
2. Then flush the flow cell with ~300 µL of 1 × TAE-Mg buffer (40 mM tris, 2 mM EDTA, 20 mM acetic acid, 12 mM MgCl_2_).

## 6. Validation of the pTIRF Setup via Typical smFRET Experiment

**Figure 8:**
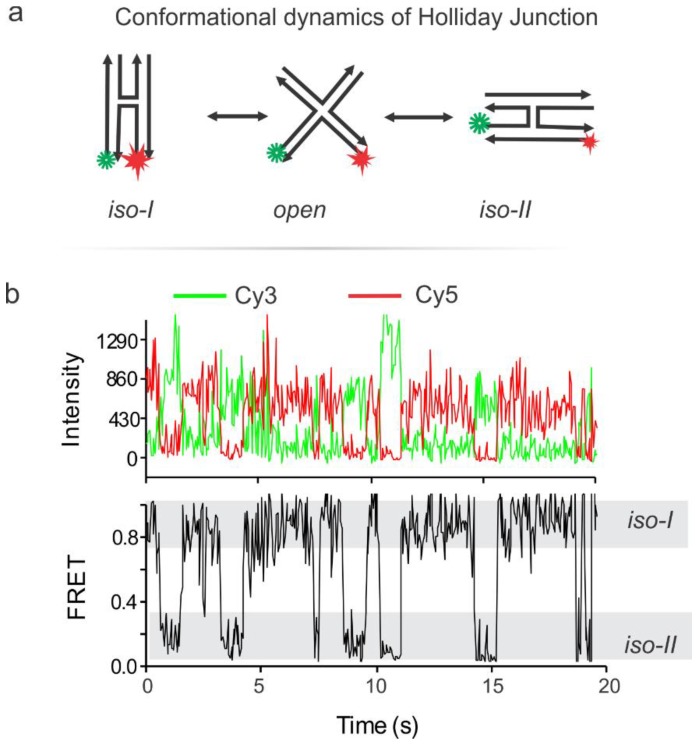
Characterization of the instrument with a typical experiment. (**a**) Conformational switching of the synthetic Holliday junction (HJ) labeled with a Cy3-Cy5 fluorophore pair. (**b**) Representative single molecule fluorescence-time traces from our smFRET experiment on the HJ. Note that the junction switches between the *Iso-I* and *Iso-II* conformations. Adapted with permission from Ref 31. Copyright 2018 American Chemical Society.

Typical smFRET data were gathered from monitoring the dynamics of the Holliday junction (HJ) [13,15,16,17]. The HJ is a central part of the double stranded DNA break repair mechanism and as such, its resolution has been seen as a possible target for drug therapy [18,19,20,21,22,23,24,25,26,27]. FRET data can be analyzed and interpreted in various ways [28,29,30,31], we used a simple efficiency analysis Equation (1). The HJ is a four-way DNA junction formed from four single-strand DNA (ssDNA). Inherent to this structure is the tendency to switch between two stacked conformation, called herein *Iso-I* and *Iso-II* (Figure 8) [32,33]. The frequency of this structural switching is dependent on the concentration of a divalent cation such as magnesium ion in the solution. For this experiment a biotinBSA/streptavidin-functionalized flow cell is treated with a biotin-functionalized, dual fluorophore labeled HJ as described in our previous work [34]. Briefly, the HJs were immersed in an imaging buffer (300 mM Mg^2+^, 40 mM Tris, 10 mM acetic acid, 1 mM EDTA, 10 mM PCA, 50 nM PCD, and 5 mM Trolox), injected into the flow cell, incubated for surface immobilization, and movies were recorded by Single.exe at a 50 ms frame rate while the green laser (532 nm) is on. The PCA, PCD and Trolox make an oxygen scavenging system (OSS) which is necessary to retard photobleaching of the fluorophores [35,36,37,38]. Typical intensity-time traces of dynamic HJ switching between *iso-I* and *iso-II* are depicted in Figure 8, showing the anti-correlation of the red and green signals typical of a FRET pair switching between a short to longer distance from one another. This is also reflected in the FRET trace calculated using Equation (1) [35,39,40].

(1) $$\mathrm{FRET}\;\mathrm{Efficiency}\;\left(E_{\mathrm{FRET}}\right)=\frac{I_{\mathrm{acceptor}}}{I_{\mathrm{donor}}+I_{\mathrm{acceptor}}}\;\left(\mathrm{eqn}\;1\right)$$

where FRET efficiency is calculated using the intensities of the donor (I_donor_) and acceptor (I_acceptor_) fluorophores. In this experiment the donor is Cy3 and the acceptor is Cy5.

## 7. Data Acquisition and Analysis

Data acquisition and analysis codes were readily available for smFRET data and were acquired upon request from the Center for the Physics of Living Cells (https://cplc.illinois.edu/software/). This package contains the data acquisition application, Single.exe which records fluorescence signal acquired by the EMCCD for each movie as a pma file. The package also includes custom written scripts for IDL and MATLAB which can be used to generate and process single molecule FRET traces from acquired pma files. The MATLAB and IDL programs are commercially available. The IDL program scripts pair molecules exhibiting fluorescence from the donor and acceptor channels and track their intensities over time. These traces can be viewed in a user friendly manner using the MATLAB program scripts available through this package. Additional MATLAB scripts for processing traces are available from various sources. We especially use scripts geared toward compiling and truncating saved molecule traces available from Fu et al., [39] which can then be graphed as FRET histograms in commercially available graphing software such as OriginPro. For more complicated data processing, particularly in cases where molecules exhibit multiple FRET states, hidden Markov Model analysis can be a useful tool to elucidate the number of states exhibited, the interconversion rates between each state, and the time spent in each state [41,42]. Hidden Markov Model analysis is available for use through the program HaMMy available from http://bio.physics.uiuc.edu/HaMMy.html.

## 8. Technical Notes

### 8.1. Prism Angle Calculation

The trapezoidal prism is positioned above the specimen chamber and the objective. The prism directs the incoming laser beam to the quartz/water TIRF interface slightly larger than the so called critical angle at which the light will be completely reflected and total internal reflection (TIR) occurs. Hence, an evanescent field is created with the same frequency as the incident light, but the intensity decays exponentially with penetration such that only fluorophores within the evanescent field are excited by the electromagnetic field and fluorescence is produced.

Snell’s law: η_1_ Sin i_a_ = η_2_ Sin i_b_

where η_1_ and η_2_ are the refractive indices of the medium 1 & 2 respectively. i_a_ & i_b_ are the angle of incident beam and the angle of refracted beam at the air/quartz prism interface. c is the critical angle.

$$c={\mathrm{Sin}}^{-1}\;\left(\frac{n_{2}}{n_{1}}\right)={\mathrm{Sin}}^{-1}\;\left(\frac{1.33}{1.55}\right)=59.1^{{}^{\circ}}$$

According to Snell’s law [40], for TIR to occur at the quartz slide/water interface, the i_2_ should be greater or equal to 59.1° which is the critical angle for the quartz slide/water interface.

**Figure 9:**
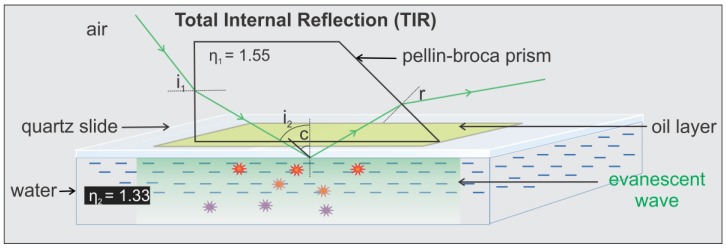
Schematic of the light path at the interface between two media in TIRFM imaging system. Refracted light at an angle of incident (i_2_) larger than the critical angle (c) undergoing total internal reflection, leading to the formation of evanescent wave. The creation of the evanescent wave allows selective excitation of fluorophores that are on or close (typically ~100 nm from the surface) [11,43,44] to the surface.

Our actual setup corresponds to Figure 9b, where i_1_ is ~35° and the calculated value of i_2_ is 68°, which is greater than the critical angle (59.1°) allowing TIR at the quartz-water interface. The depths of penetration (d) calculated using the equation [11,43,44] below are 78 and 94 nm for the green (λ = 532 nm) and red (λ = 639 nm) lasers, respectively.

$$d=\frac{\lambda }{4\pi }{\left(\eta_{1}^{2}\sin^{2}i_{2}-\eta_{2}^{2}\right)}^{-1/2}$$

where λ is the wavelength of the incident light in vacuum. The depth of penetration relies on i_2_ (ultimately i_1_), wavelength of the incident light, and refractive indices of the mediums, however it has been demonstrated that it is independent of the polarization of the incident light [11].

### 8.2. Prism Clamp

**Figure 10:**
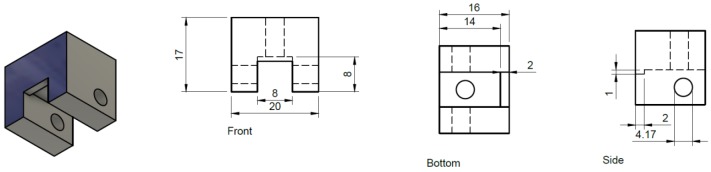
Dimensions of prism clamp from three directions. The sides are mirrored and the holes taped with 8–32 machine threading. All of the dimensions are in millimeter (mm).

The basic function of the prism clamp is to hold the prism in place and to provide a way to mount it to the prism support arm (described below). Our design has proven very reliable but any device which can accomplish the task would be suitable. Our prism clamp was designed in house and the design of the clamp is detailed in Figure 10. Our clamp was machined out of aluminum in Virginia Commonwealth University’s on-campus machining shop. Off the shelf 8–32 machine screws from a hardware store were used to tighten the prism into place. It is important to remember that our clamp is dimensioned to a specific prism (part#:325–1206 UV FS Pellin-Broca Prism 11 mm × 20 mm × 6.4 mm), the design would need to be adapted for any other size of prism. While we chose to make the clamp out of aluminum for durability a 3D printed part with some modifications to insert nuts into the sidewalls of the clamp would also be a viable design. 

### 8.3. Prism Support Arm

**Figure 11:**
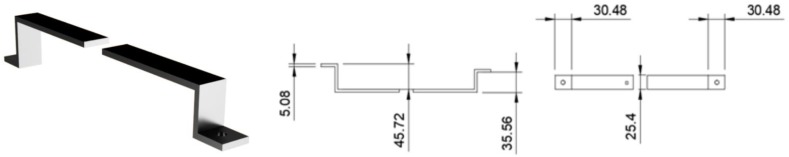
Dimensioned drawing of prism support arm from 3D printer file. **Left**, a 3D rendering of the finished item; Middle, side view; **Right**, top view. Units are in mm, round holes are 7.6 mm wide, square hole is 5 mm.

The prism support arm (Figure 11) provides a mount for the prism clamp, we designed ours to work with our microscope but the dimensions could be altered to suit others. Our arm was printed out of PLA plastic on a MakerBot replicator (5th generation), stl files will be made available upon request. Due to dimensional limitations of our printer the support arm was printed in two pieces and then glued together. We would recommend producing the arm as a single piece for durability if large enough printer is available or if machining the arm out of metal. It is also ideal to print the part in a matte black material to avoid potential laser scattering. The exact placement and size of the square center hole needed for the screw shaft to mount the prism clamp is dependent on the dimensions of the set up and where the prism needs to be mounted to achieve TIR. It is advised to use a long thin rectangular channel allowing space for the prism holder to be adjusted closer and further from the focusing lens.

## 9. Conclusions

Here we describe the detail guidelines for building the prism-based TIRF microscope using commercially available microscope parts. Splitting the microscope setup procedure into three sections, (a) excitation path(s), (b) focusing and beam positioning, and (c) emission path, we presented stepwise instructions on building and characterizing a pTIRF instrument incorporating necessary precautions whenever necessary. Once the instrument is ready, in order to check its operation, we outlined flow cell design and its surface-functionalization, followed by conducting a representative pTIRF experiment using a dual-labeled DNA HJ. After data acquisition and analysis with the aid of MATLAB scripts and OriginPro, we saw clear conformation switching of HJ, thus demonstrating the successful designing and functioning of our pTIRF microscopy. Although we tested our instrument with the HJ, this instrument is equally applicable to reveal dynamics, kinetics, and other structure/conformation related information of other biomolecules, such as RNA and proteins.

## Figures and Tables

**Table 1:** Extensive list of microscope parts along with the catalog numbers, parts descriptions, and the vendors from which we bought the parts to build our microscope. The numbers in the rightmost column correspond to the optics numbering used in the Figures.

Catalog/Item #	Item Description	Quantity	Vendor	Optics #
p/n 325–1206	p/n 325–1206 UV FS Pellin-Broca Prism 11 × 20 × 6.4 mm	2	Altos Photonics, Inc. (Bozeman, MT, USA )	16
	Laser Barrier 150” wide X 92” long	1	Beamstop’r (Lyndhurst. OH, USA	
16212	Immersion Oil Type FF (4 Fl. Oz.)	2	Cargille (Cedar Grove, NJ, USA)	
zet640/20x	magnetron bandpass clean-up filter (excitation path), 0–5 deg aoi	1	Chroma Technology Corp (Bellows Falls, VT, USA)	5
t565spxxr-uf3	magnetron shortpass dichroic, 45 deg aoi	1	Chroma Technology Corp	6
1069417	SYS: CUBE 640-40 CIRCULAR: 640 nm: 40 mW	1	Coherent Inc. (Santa Clara, CA, USA)	2
1073840	ASSY: HEAT SINK: ACCESSORY: CUBE	1	Coherent Inc.	
1214333	Productivity Plus Bronze-CUBE	3	Coherent Inc.	
EW-06419-01	Tygon Microbore Autoanalysis Tubing, 0.020” × 0.060”OD, 100 ft/roll	4	Cole-Parmer	
CL532-050-L	532 nm Central Wavelength 50 mW CW Power	1	CyrstaLaser (Reno, NV, USA)	1
4001	Hardman DOUBLE/BUBBLE Extra-Fast Set Epoxy Red Package 3.5 g Packet	3	Ellsworth Adhesive Systems (Germantown, WI, USA)	
KBH-5503	Laser Protective Eyewear for HeNe Alignment and KTP Alignment applications	2	Kentek Corporation (Pittsfield, NH, USA)	
9470	15 ft USB 3.0 A Male to A Female Active Extension Cable	2	Monoprice (Rancho Cucamonga, CA, USA)	
5010	Cat6 24AWG UTP Ethernet Network Patch Cable, 20 ft Gray	2	Monoprice	
2067	USB to RS232 DB9 male(Serial)/DB25 male Converter Cable	1	Monoprice	
U-R380	IX3-D6RES;6-POSITION IX NOSEPIECE CODED, DIC	1	Olympus America Inc. (Center Valley, PA, USA)	
U-V111C	U-TV1XC;C-MOUNT CAMERA ADAPTER, CENTERABLE	1	Olympus America Inc.	
9-U734	45FR; 45MM FROSTED DIFFUSION FILTER, IX3	1	Olympus America Inc.	
UYCP-11	UYCP-11;US STYLE 3-PRONG POWER CORD	1	Olympus America Inc.	
5-UR403	IX3-RFA;STRAIGHT ILLUMINATOR	1	Olympus America Inc.	17
5-UR416-1	IX3-RFACS-1-2; CODED IX3FLUORESCENCE TURRET	1	Olympus America Inc.	17
OCT-TD7BX3	TRF59907-OL3; Dual-band ET-532/640 nm laser TIRF set	1	Olympus America Inc.	
OAT-DU-897U-CS0-#BV	DU-897U-CS0-#BV; IXON ULTRA897 EMCCD, 56FPS, 512 × 512, 16 UM, USB	1	Olympus America Inc.	19
OAT-TR-EMFS-F06	532/640 EM SPLITTING 3 PART FILTER SET FOR DPC&OPTOSPLIT	1	Olympus America Inc.	
OAT-TR-DCIS-CA1-00	DUAL CAM CASSETTE. REQ(TR-EMF S-F)	1	Olympus America Inc.	
O89-OptoIILS	OptoSplit II LS–1.0x; Optosplit II system w/cubes and diaphragm	1	Olympus America Inc.	18
OMT-010	MT-010; LOGO CLOTH DUSTCOVER 11 × 25 × 26”, ANTI-STATIC BX, IX	1	Olympus America Inc.	
OVP-MSTUT2468	M-ST-UT2-46-8; Tuned-Damped Table, 1200 × 1800 × 203 mm, M6 Holes	1	Olympus America Inc.	
OVP-S2000A428	S-2000A-428; 28” Isolators w/Auto Leveling, Set of 4	1	Olympus America Inc.	
OVP-ACWS	ACWS; Air Compressor, Low Noise, 110 V	1	Olympus America Inc.	
HPZ440WIN7-2	2805181;HP Z440, 2 × 1TBHD, RAID1, 32GB DDR3, WIN764, SERIAL, MS OFC	1	Olympus America Inc.	
D-M27FPW2	718668226; 27-INCH 16:9 RATIO FLAT PANEL, 4K UHD, HDMI, D PORT	1	Olympus America Inc.	
DIB-551.00	Diamond Coated “Stick” Drills, DIB-551.00	10	Shor International	
TR6-P5	Ø1/2” Optical Post, SS, 8-32 Setscrew, 1/4”−20 Tap, L = 6”, 5 Pack	3	Thorlabs, Inc. (Newton, NJ USA)	
MB1218	Aluminum Breadboard 12” × 18” × 1/2”, 1/4”−20 Taps	1	Thorlabs, Inc.	
SH8S050	8-32 Stainless Steel Cap Screw, 1/2” Long	1	Thorlabs, Inc.	
TR12	Ø1/2” Optical Post, SS, 8-32 Setscrew, 1/4”−20 Tap, L = 12”	6	Thorlabs, Inc.	
SH25S038	1/4”-20 Stainless Steel Cap Screw, 3/8” Long	1	Thorlabs, Inc.	
RA90	Right-Angle Clamp for Ø1/2” Posts, 3/16” Hex	8	Thorlabs, Inc.	
TR6	Ø1/2” Optical Post, SS, 8-32 Setscrew, 1/4”−20 Tap, L = 6”	1	Thorlabs, Inc.	
TR075	Ø1/2” Optical Post, SS, 8-32 Setscrew, 1/4”−20 Tap, L = 0.75”	2	Thorlabs, Inc.	
SS25S075	1/4”−20 Stainless Steel Setscrew, 3/4” Long, Pack of 25	1	Thorlabs, Inc.	
SH25S075	1/4”−20 Stainless Steel Cap Screw, 3/4” Long, Pack of 25	1	Thorlabs, Inc.	
B3648F	36” × 48” × 2.4” Imperial Breadboard, 128 × 98 × 23 cm	1	Thorlabs, Inc.	
PSY313	900 × 1200 mm Full Under Shelf, 146 × 95 × 6 cm	1	Thorlabs, Inc.	
PTA512	Air Compressor-110/115 V-60 Hz, US Power Plug, 45 × 38 × 46 cm	1	Thorlabs, Inc.	
RSP1	Rotation Stage For 1” Optics 2.2”OD 1.062-20 ID	1	Thorlabs, Inc.	
TR3-P5	1/2” Dia. × 3” Length: Pack of 5 Post	3	Thorlabs, Inc.	
SS6MS25	M6-1.0 × 25 mm Set Screw, 25 Pack	2	Thorlabs, Inc.	
SS6MS12	M6 × 1.0 Stainless Steel Set Screw 12 mm Long Pack of (25)	1	Thorlabs, Inc.	
FMP1-P5	Fixed Ø1” Optical Mount 5-Pack	2	Thorlabs, Inc.	
WP25M-VIS	Mounted Ø25.0 mm Wire Grid Polarizer, 420–700 nM	1	Thorlabs, Inc.	4
ID8	Mounted Standard Iris, 8.0.mm max. Aper.	1	Thorlabs, Inc.	7
ID12	Iris Diaphragm 1/2”	1	Thorlabs, Inc.	
ID15	Mounted Standard Iris, 15.0.mm max. Aper.	1	Thorlabs, Inc.	9
WPMH05M-532	Mounted Multi Order 1/2 Waveplate 532 nm	1	Thorlabs, Inc.	3
BB1-E02	Ø25.4 mm Mirror, Broadband 400–750 nm	4	Thorlabs, Inc.	10–13
LB1904-A-ML	Mounted N-BK7 Bi-Convex Lens, Ø1”, f = 125 mm, -A	1	Thorlabs, Inc.	
LB1437-A-ML	Mounted N-BK7 Bi-Convex Lens, Ø1”, f = 150 mm, -A	1	Thorlabs, Inc.	
LB1945-A-ML	Mounted N-BK7 Bi-Convex Lens, Ø1”, f = 200 mm, -A	1	Thorlabs, Inc.	14
ESK01	MOUNTING SUPPORTS ESSENTIALS KIT #1	1	Thorlabs, Inc.	
LG1	Laser Glasses, 190–400 nm, 808–1090 nm	1	Thorlabs, Inc.	
ADB-10	Pellin Broca Prism 10 mm BK7	1	Thorlabs, Inc.	16
MT3A/M	XYZ Metric Translator Stage	1	Thorlabs, Inc.	15
KS1	Lockable Kinematic 1” Optic Mount	4	Thorlabs, Inc.	
SDA90120S	Standing Height Active Science Desk to suit 900 × 1200 mm	1	Thorlabs, Inc.	
LS6S2T0	Uni-stabled housed Shutter	1	Vincent Associates	8
VCM-D1	Shutter Driver	1	Vincent Associates	
